# Supplementary material for: Synaptotagmin 13 Is Highly Expressed in Estrogen Receptor-Positive Breast Cancer
Source: Curr Oncol. 2021 Oct 12;28(5):4080–92. doi: 10.3390/curroncol28050346 (PMC8535095; doi:10.3390/curroncol28050346)
Supplement: Supplementary file 1 [file curroncol-28-00346-s001.zip › curroncol-1405597-supplementary.pdf]

Supplementary Table S1.

SYT13 mRNA expression levels of 58 BC cell lines in the CCLE database.

Cell Line Name	ER Status	PgR Status	Relative *SYT13* mRNA Expression Level (log2 (TPM+1))
AU565	–	–	0.475084883
BT20	–	–	0.028569152
BT474	+	+	0.250961574
BT483	+	+	2.702657543
BT549	–	–	0.097610797
CAL120	–	–	0.070389328
CAL148	–	–	2.757023247
CAL51	–	–	0.042644337
CAL851	–	–	0.028569152
CAMA1	+	+	0.782408565
DU4475	–	–	3.707082992
EFM19	+	+	3.032100843
EFM192A	+	+	1.906890596
HCC1143	–	–	0.765534746
HCC1187	–	–	0.678071905
HCC1395	–	–	0.189033824
HCC1419	+	–	0.014355293
HCC1428	+	+	1.321928095
HCC1500	–	–	3.499527024
HCC1569	–	–	0.124328135
HCC1599	–	–	0.028569152
HCC1806	–	–	0.070389328
HCC1937	–	–	0.443606651
HCC1954	–	–	0.344828497
HCC202	–	–	0.014355293
HCC2157	–	–	0
HCC2218	–	–	0.042644337
HCC38	–	–	1.117695043
HCC70	–	–	0.014355293
HDQP1	–	–	0.056583528
HS578T	–	–	0.111031312
JIMT1	–	–	0.028569152
KPL1	–	–	0.622930351
MCF7	+	+	0.831877241

MDAMB134VI	+	–	2.060047384
MDAMB157	–	–	0.137503524
MDAMB175VII	+	–	0.263034406
MDAMB231	–	–	0.111031312
MDAMB361	+	–	1.14404637
MDAMB415	+	–	3.049630768
MDAMB436	–	–	0.097610797
MDAMB453	–	–	0.028569152
MDAMB468	–	–	0.411426246
MFM223	–	–	0
SKBR3	–	–	0.084064265
SUM102PT	–	–	0.014355293
SUM1315MO2	–	–	0
SUM149PT	–	–	0
SUM159PT	–	–	0.014355293
SUM185PE	–	–	0.056583528
SUM229PE	–	–	0.070389328
SUM44PE	+	–	1.10433666
SUM52PE	+	–	0
T47D	–	+	0.201633861
UACC812	+	+	0.807354922
UACC893	–	–	1.469885976
ZR751	+	–	2.032100843
ZR7530	+	–	0.757023247

BC, breast cancer; CCLE, cell line encyclopedia; ER, estrogen receptor; PgR, progesterone receptor; TPM, transcripts per million.

Supplementary Table S2.

Correlations between mRNA expression levels of *SYT13* and 84 cancer-related genes.

Gene	Official Full Name	Correlation Coefficient	*P*-value
ABL1	ABL proto-oncogene 1, non-receptor tyrosine kinase	0.4615	0.1124
AKT1	AKT serine/threonine kinase 1	0.6813	0.0103
APC	APC regulator of WNT signaling pathway	0.6538	0.0153
ATM	ATM serine/threonine kinase	0.2692	0.3737
BAX	BCL2 associated X, apoptosis regulator	0.3352	0.2629
BCL2	BCL2 apoptosis regulator	0.6703	0.0122
BCL2L1	BCL2 like 1	–0.1264	0.6808
BCR	BCR activator of RhoGEF and GTPase	–0.1868	0.5411
BRCA1	BRCA1 DNA repair associated	0.6429	0.0178
BRCA2	BRCA2 DNA repair associated	0.6484	0.0165
CASP8	caspase 8	0.1264	0.6808
CCND1	cyclin D1	0.5055	0.078
CDH1	cadherin 1	0.5659	0.0438
CDK4	cyclin dependent kinase 4	0.5989	0.0306
CDKN1A	cyclin dependent kinase inhibitor 1A	0.5275	0.064
CDKN2A	cyclin dependent kinase inhibitor 2A	0.3681	0.2159
CDKN2B	cyclin dependent kinase inhibitor 2B	0.3714	0.2115
CDKN3	cyclin dependent kinase inhibitor 3	0.4835	0.0941
CTNNB1	catenin beta 1	–0.1429	0.6415
E2F1	E2F transcription factor 1	0.1429	0.6415
EGF	epidermal growth factor	0.3901	0.1876
ELK1	ETS transcription factor ELK1	0.2198	0.4706
ERBB2	erb-b2 receptor tyrosine kinase 2	0.1374	0.6545
ESR1	estrogen receptor 1	0.6538	0.0153
ETS1	ETS proto-oncogene 1, transcription factor	–0.2802	0.3538
FHIT	fragile histidine triad diadenosine triphosphatase	0.2747	0.3637
FOS	Fos proto-oncogene, AP-1 transcription factor subunit	0.2088	0.4936
FOXD3	forkhead box D3	–0.0549	0.8585
HGF	hepatocyte growth factor	0.0221	0.9429
HIC1	HIC ZBTB transcriptional repressor 1	–0.1264	0.6808
HRAS	HRas proto-oncogene, GTPase	0.1209	0.694
IGF2R	insulin like growth factor 2 receptor	–0.0824	0.789
JAK2	Janus kinase 2	0.2308	0.4481
JUN	Jun proto-oncogene, AP-1 transcription factor subunit	–0.1758	0.5656

JUNB	JunB proto-oncogene, AP-1 transcription factor subunit	0.2363	0.4371
JUND	JunD proto-oncogene, AP-1 transcription factor subunit	–0.1484	0.6286
KIT	KIT proto-oncogene, receptor tyrosine kinase	0.7253	0.005
KITLG	KIT ligand	0.4176	0.1557
KRAS	KRAS proto-oncogene, GTPase	0.7912	0.0013
MCL1	MCL1 apoptosis regulator, BCL2 family member	0.2473	0.4154
MDM2	MDM2 proto-oncogene	0.7967	0.0011
MEN1	menin 1	0.4066	0.168
MET	MET proto-oncogene, receptor tyrosine kinase	–0.2363	0.4371
MGMT	O-6-methylguanine-DNA methyltransferase	0.456	0.1173
MLH1	mutL homolog 1	0.4615	0.1124
MOS	MOS proto-oncogene, serine/threonine kinase	0.2967	0.3249
MYB	MYB proto-oncogene, transcription factor	0.7088	0.0067
MYC	MYC proto-oncogene, bHLH transcription factor	0.0659	0.8305
MYCN	MYCN proto-oncogene, bHLH transcription factor	0.6099	0.0269
NF1	neurofibromin 1	0.5934	0.0325
NF2	neurofibromin 2	0.4176	0.1557
NFKB1	nuclear factor kappa B subunit 1	0.5385	0.0576
NFKBIA	NFKB inhibitor alpha	0.7253	0.005
NRAS	NRAS proto-oncogene, GTPase	0.2967	0.3249
PIK3C2A	phosphatidylinositol-4-phosphate 3-kinase catalytic subunit type 2 alpha	0.478	0.0985
PIK3CA	phosphatidylinositol-4,5-bisphosphate 3-kinase catalytic subunit alpha	0.5055	0.078
PML	PML nuclear body scaffold	0.011	0.9716
PRKCA	protein kinase C alpha	0.0055	0.9858
RAF1	Raf-1 proto-oncogene, serine/threonine kinase	0.3736	0.2086
RARA	retinoic acid receptor alpha	0.6374	0.0191
RASSF1	Ras association domain family member 1	–0.1648	0.5905
RB1	RB transcriptional corepressor 1	0.2527	0.4048
REL	REL proto-oncogene, NF-kB subunit	0.4176	0.1557
RET	ret proto-oncogene	0.4176	0.1557
ROS1	ROS proto-oncogene 1, receptor tyrosine kinase	–0.0165	0.9574
RUNX1	RUNX family transcription factor 1	0.3297	0.2713
RUNX3	RUNX family transcription factor 3	0.1593	0.6031
S100A4	S100 calcium binding protein A4	0.2253	0.4593
SERPINB5	serpin family B member 5	0.2747	0.3637
SH3PXD2A	SH3 and PX domains 2A	–0.1538	0.6158

SMAD4	SMAD family member 4	0.3736	0.2086
SRC	SRC proto-oncogene, non-receptor tyrosine kinase	0.2088	0.4936
STAT3	signal transducer and activator of transcription 3	0.2143	0.4821
STK11	serine/threonine kinase 11	0.1484	0.6286
TGFB1	transforming growth factor beta 1	0.1099	0.7208
TNF	tumor necrosis factor	0.011	0.9716
TP53	tumor protein p53	0.2198	0.4706
TP73	tumor protein p73	0.6648	0.0132
TSC1	TSC complex subunit 1	0.2692	0.3737
VHL	von Hippel-Lindau tumor suppressor	0.3462	0.2466
WT1	WT1 transcription factor	0.0934	0.7615
WWOX	WW domain containing oxidoreductase	0.6813	0.0103
XRCC1	X-ray repair cross complementing 1	0.6813	0.0103
ZHX2	zinc fingers and homeoboxes 2	0.4451	0.1275
